# Early considerations for village-in-a-dish platform: a multidisciplinary perspective

**DOI:** 10.3389/fbioe.2026.1923405

**Published:** 2026-09-14

**Authors:** Talha Qureshi, Anna Bertocci, Ken Redekop, Pieter Vandekerckhove, Maarten IJzerman, Joyce van Meurs, Carin Uyl-de Groot, Roberto Narcisi

**Affiliations:** 1 Delft Centre for Entrepreneurship, Erasmus School of Health Policy and Management, Erasmus University Rotterdam, Rotterdam, Netherlands; 2 Department Orthopaedics and Sports Medicine, Erasmus Medical Centre, Rotterdam, Netherlands; 3 Delft Centre of Entrepreneurship, TU Delft, Delft, Netherlands; 4 Department of Internal Medicine and Orthopaedics and Sports Medicine, Erasmus Medical Centre, Rotterdam, Netherlands

**Keywords:** conventional cell culture, genetic variation, HTA core model, induced pluripotent stem cells, personalized medicine, single-cell RNA sequencing, village-in-a-dish

## Abstract

The conventional cell culture models are not suitable for large-scale studies of genetic variation in disease mechanisms and drug response due to technical variability, high cost, and labor associated with them. Multiplex cell culture approaches, such as Village-in-a-Dish (ViaD), can address these limitations by enabling simultaneous studies of cells from many individuals in a single experiment. However, the multiplex design of ViaD poses several issues that differ from those of conventional cell culture. In this review, we identify and expound on the issues with ViaD and categorized them as platform-related (inherent to the design) or application-related (dependent on specific context or use). By providing a structured overview of these issues, our findings can help researchers incorporate key technical considerations during study design and interpretation and determine the context in which the multiplex cell culture approaches might be unsuitable.

## Introduction

Advances in human cell culture technologies have transformed biomedical research by enabling increasingly precise modelling of biological processes *in vitro*. In particular, induced pluripotent stem cells (iPSCs), first generated from human somatic cells by Takahashi and Yamanaka in 2007 (Takahashi et al., 2007), have become central to *in vitro* studies of genetic variation, disease mechanisms, and drug responses (Cerneckis et al., 2024; Grant Rowe and Daley, 2019; Spitalieri et al., 2016; Yamanaka, 2026).

The ability to generate patient and donor-specific iPSCs, combined with their capacity to self-renewal and differentiation into diverse cell types, provided a powerful framework to study human genetic diversity *in vitro*. These approaches have been especially successful in studies of monogenic disorders, where high-impact mutations produce relatively clear and interpretable cellular phenotypes. However, modelling complex polygenic diseases and traits remains challenging, as it requires experimental designs that capture inter-individual genetic heterogeneity at scale (Boyle et al., 2017; Ghosh et al., 2022; van Zanten et al., 2026).

Conventional cell culture approaches face several limitations in this context. Studying large numbers of donor-derived cell lines in parallel is labor-intensive, costly, and prone to technical variability arising from differences in batch and operator effects. Furthermore, iPSC-based models can be influenced by additional sources of biological and technical variation, including clone-to-clone variability, genomic instability acquired during prolonged culture, and differences in lineage commitment, maturation, and differentiation efficiency between donor lines. These issues complicate efforts to disentangle the true genetic effects from the experimental noise (Jerber et al., 2021).

Consequently, there is growing interest in experimental platforms that enable high-throughput, standardized analysis of donor-specific cellular responses while reducing technical and organizational burdens. The technological advances have progressively enabled the transition from studying individual donor-derived iPSC lines towards population-scale cellular studies. The establishment of large-scale iPSC biobanks has provided access to hundreds or thousands of genetically diverse donor-derived iPSC lines, creating unprecedented opportunities to investigate the relationship between genetic variation and cellular phenotypes (Kilpinen et al., 2017; Panopoulos et al., 2017). In parallel, advances in single-cell technologies, high-throughput sequencing, and computational approaches for genetic demultiplexing have enabled the identification of individual donor contributions within pooled cellular experiments. Together, these developments have made it possible to combine multiple genetically distinct donor-derived cell lines within a single experimental system, while retaining donor-specific resolution.

The Village-in-a-Dish (ViaD) is a relatively new cell culture approach where cells from many donors are pooled together in a single dish and analyzed via single-cell sequencing techniques. It differs from conventional cell culture, where cells from individual donors are cultured and analyzed separately using either single-cell or bulk sequencing technologies (Figure 1). ViaD builds directly on the aforementioned technological advances and represents a recent and innovative response to minimize the technical variability, costs, time, and labor associated with large-scale polygenic studies (Farbehi et al., 2024; Neavin et al., 2023; Wells et al., 2023).

**FIGURE 1:**
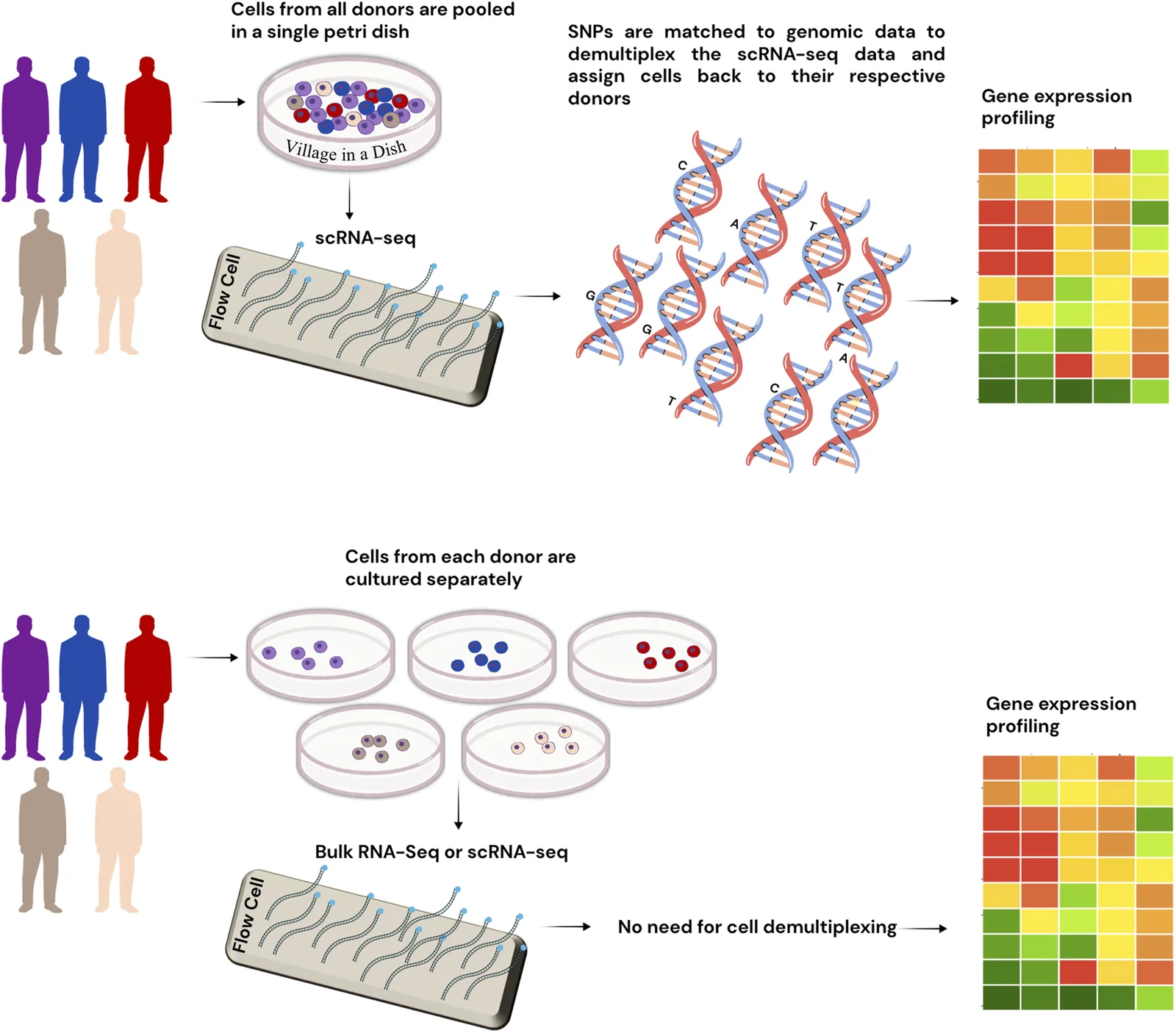
Overview of Village-in-a-Dish (ViaD) workflow compared with conventional cell culture. In ViaD (top), cells from multiple donors are pooled in a single petri dish, analyzed via scRNA-Seq and computationally demultiplexed using single nucleotide polymorphisms (SNPs) to recover donor identities for gene expression profiling. In conventional cell culture (bottom), cells from each donor are cultured and analyzed separately via bulk-RNA or scRNA-Seq without demultiplexing.

Nevertheless, the very features that make ViaD a powerful tool also introduce new issues and limitations. As ViaD approach is relatively new and is not yet widely adopted, many of these issues have not yet been systematically recognized, leaving researchers uncertain not only about its implementation but also about the types of biological questions for which ViaD is most appropriate. Therefore, in this review we aim to identify and discuss the broader issues and limitations associated with ViaD that are unique to its multiplex design and those that are also present in conventional single-donor cultures but are amplified in ViaD. We further categorize the identified issues as platform-related (inherent to the design) or application-related (dependent on specific context or use) (Figure 2).

**FIGURE 2:**
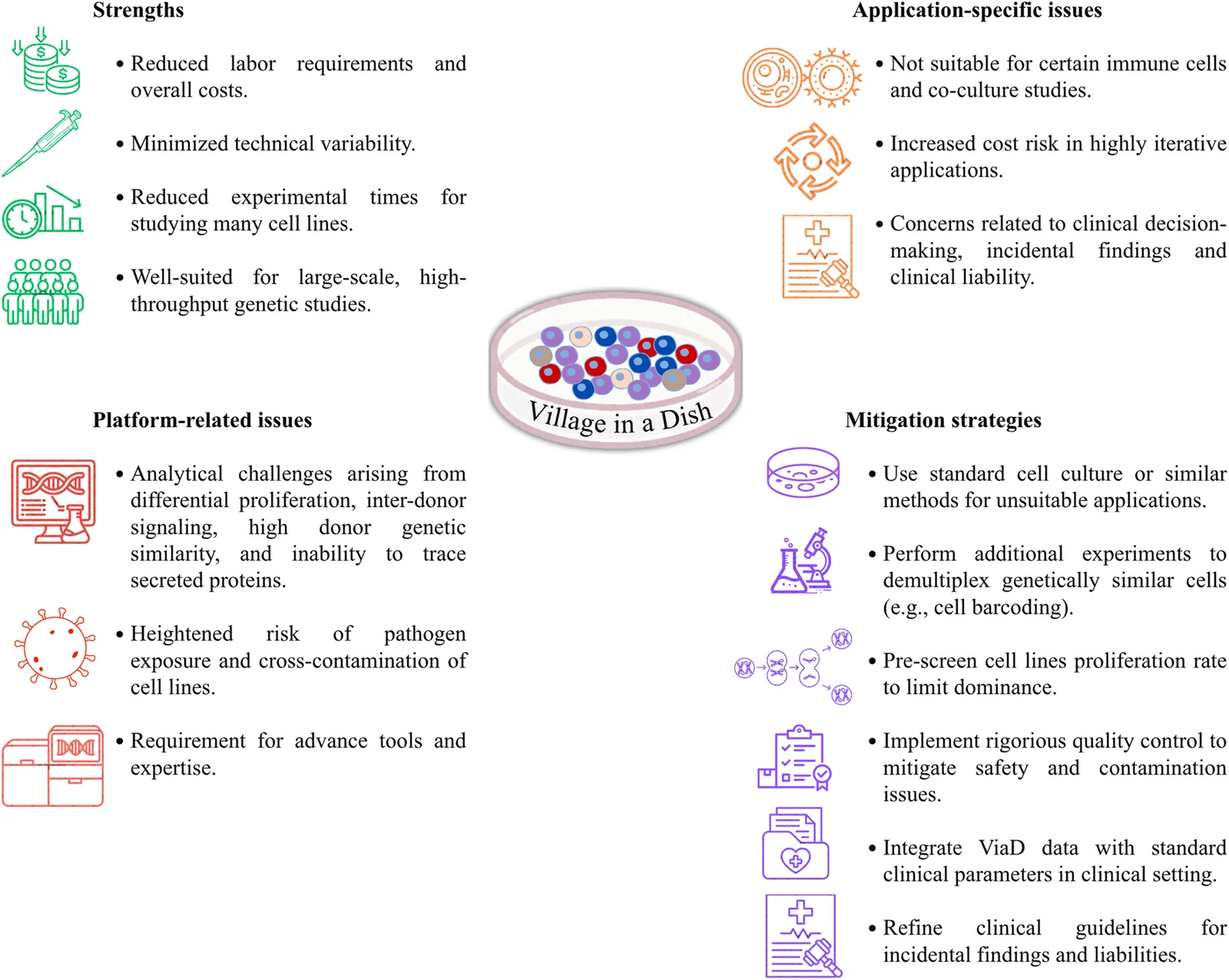
Strengths, issues and mitigation strategies of Village-in-a-Dish (ViaD) platform. ViaD reduces, labor, costs, time and technical variability compared to conventional cell culture and is well-suited for large-scale genetic studies. Platform-related issues and application-specific issues outline analytical, contamination, cell type and clinical-translation challenges associated with the pooled-culture approach. Corresponding mitigation strategies are proposed to address each category of limitation.

To do so, we draw on elements of the health technology assessment (HTA) Core Model to organize early considerations relevant to ViaD across technical, practical and societal domains. HTA is traditionally used to evaluate mature health technologies for their clinical value across multiple domains, including technical characteristics, safety, clinical aspects, organizational aspects, ethical considerations, and economic implications (Kristensen et al., 2017; Maarten and Steuten, 2011; O'Rourke et al., 2020). Here, these domains are not used to assess clinical value. Instead, they are applied selectively as an analytical lens to structure reflection on ViaD as an emerging *in vitro* cell culture platform.

### ViaD as an emerging multiplex cell culture platform

The ViaD approach is an *in vitro* cell culture platform designed to enable population-scale analysis of donor-specific cellular phenotypes within a shared experimental environment.

At the technical level, ViaD typically relies on iPSCs or differentiated cell types that share the same tissue identity. Prior to pooling, donor-specific cell lines are expanded and characterized, after which they are combined in defined proportions to form a “village”. Following exposure to stimuli such as drugs, environmental stressors, or differentiation cues, cells are analyzed using scRNA-seq or related readouts. Demultiplexing tools enable the assignment of individual cells back to their respective donors, allowing inter-individual variability in gene expression, cellular responses, and differentiation trajectories to be studied within a single experiment (Mitchell et al., 2020; Neavin et al., 2023; Wells et al., 2023).

This design offers several advantages over conventional cell culture approaches. By analyzing cell lines from multiple donors simultaneously, potentially scaling to hundreds, ViaD reduces batch effects, experimental variability, cost and labor associated with maintaining parallel cultures. It also enables the study of genetic heterogeneity at a scale that would be impractical using traditional methods, facilitating population-level investigations of disease mechanisms, viral susceptibility, and cellular differentiation processes (Cederquist et al., 2020; Wells et al., 2023). As such, ViaD has attracted considerable interest as a platform for studying complex, polygenic traits and for generating data relevant to functional precision medicine.

However, the pooled nature of ViaD also introduces technical and interpretative issues that are not encountered in conventional cell culture. Taken together, these features position ViaD as a powerful experimental platform, whose strengths and limitations are highly context dependent.

Therefore, decisions regarding the experimental design, choice of biological questions, and interpretation of results can substantially influence the robustness and relevance of findings generated using ViaD.

### A domain-based framework for structuring considerations in early-stage platform evaluation

Emerging *in vitro* platforms, such as ViaD, present biomedical researchers with a complex mix of technical opportunities, experimental constraints, and contextual uncertainties. Although many of these issues are eventually recognized, they are often identified only during experiments or data analysis rather than considered beforehand. A structured framework helps researchers identify these issues early, improving experimental design and the interpretation of results. Table 1 summarizes the domains used in this review to structure these considerations.

**TABLE 1:** Overview of domains used to structure early-stage considerations for the ViaD platform.

Domain	What this domain captures (biology-relevant)	Relevance for ViaD as a platform
Technical and Research domain	Core design features of the platform	Pooled donor cultures, demultiplexing constraints
Technical and Research domain	Ability to capture biologically meaningful variation	Detecting donor-specific effects under shared culture conditions
Practical domain	Biosafety and experimental feasibility	Contamination risk amplified by pooled cultures
Practical domain	Factors affecting feasibility at scale	Upfront setup costs; risk of whole-culture loss
Practical domain	Expertise and infrastructure requirements	Need for integrated wet lab and bioinformatics workflows
Societal domain	Limited physiological representation	Caution in translation of results to patients
Societal domain	Liabilities, and data governance	Increased data privacy concern

*The domains used to structure early-stage considerations for ViaD illustrates how different types of biological, technical, and contextual issues are organized prior to detailed mapping of platform-level and application-specific issues.

The domains considered in this framework include.

#### Technical and research domain

The technical and research domain encompasses core design features of ViaD and its ability to capture biologically meaningful variation relevant to the research question. These include pooled donor cultures, differential growth rates, inter-donor interactions, demultiplexing strategies and analytical readouts, and the ability to detect donor-specific effects and model genetic heterogeneity. Understanding these characteristics early in the study design can help distinguish biologically meaningful variations from experimental artifacts and identify potential limitations of the platform and experimental design.

#### Practical domain

The practical domain includes the feasibility and implementation of ViaD in laboratory settings. These include laboratory biosafety management in pooled cultures, coordination between wet-lab scientists and bioinformaticians, access to single-cell sequencing infrastructure and computational pipelines, and cost drivers such as upfront setup costs and the risk of entire culture failure. Considering these factors early in the study design can help anticipate resource requirements, identify barriers to implementation, and establish stringent quality control measures and safety procedures.

#### Societal domain

The societal domain covers the interface between ViaD and its broader implications for clinical translation, as well as legal and ethical considerations. Key aspects include the relevance of ViaD findings for clinical decision-making, the potential for clinically significant incidental findings, Early consideration of these aspects can enable proactive planning for clinical data translation and inform the development of frameworks to address liability and privacy concerns.

In the following section, we use this domain-based framework to systematically map platform-level and application-specific issues associated with ViaD. By doing so, we aim to illustrate how structured cross-domain thinking can support early experimental decision-making for multiplex cell culture platforms before specific downstream applications are fully defined.

### Approach to identifying platform-level and application-specific issues

This review adopts a structured, domain-based approach to identify and organize issues associated with the ViaD platform using HTA core model (version 3.0).

The issues associated with ViaD were identified through three complementary sources. First, a targeted literature review was conducted focusing on multiplex cell culture systems, population-scale iPSC studies, single-cell technologies, and early assessment of health technologies. Second, theoretical extrapolation was used to anticipate issues based on established biological principles such as cell-cell signaling, immune recognition, and technical constraints of pooled culture systems. Third, insights from interdisciplinary discussions with researchers experienced in wet-lab biology, bioinformatics, and translational research informed the identification and interpretation of potential issues.

### Mapping platform-level and application-specific issues of ViaD

Using the domain-based framework outlined above, issues associated with the ViaD platform were systematically identified and categorized across technical, biological, organizational, social, and scalability-related domains. To preserve conceptual clarity, the issues were explicitly distinguished as either platform-level, arising from the intrinsic multiplex design of ViaD and are applicable across all uses of ViaD, or application-specific, emerging only in particular experimental or translational contexts.

An overview of platform-level issues is provided in Table 2, while application-specific issues are summarized in Table 3. Some issues are unique to ViaD, while others also occur in conventional cell cultures but are amplified in ViaD setting. A third category (classified here as overlapped) comprises issues that are equally relevant to conventional cell culture in principle, but are more likely to be encountered in ViaD, given its anticipated future implementation in clinical contexts due to expected reduced costs, labor, and time (Tables 2,3).

**TABLE 2:** Overview of platform-related issues across domains.

Domain	Issue	Unique to ViaD/Amplified/Overlapped	Notes	Reference
Technical and Research domain	Differences in proliferation, differentiation or survival rates can lead to unequal donor representation	Unique to ViaD	Inherent to ViaD design, as fast-growing cell lines may dominate the dish in every application	(Neavin et al., 2023)
Technical and Research domain	Paracrine cell signaling between cells of different donors can complicate data interpretation	Unique to ViaD	Inherent to ViaD design, as cell signaling between cells of different donors can influence each other and it occurs in all applications	(Neavin et al., 2023)
Technical and Research domain	Genetically identical or highly similar lines cannot be demultiplexed easily	Unique to ViaD	ViaD relies on naturally occurring genetic variation to assign cells to their donor of origin. Consequently, genetically identical or highly similar lines may require additional demultiplexing approaches	(Cuomo et al., 2020) Theoretical extrapolation based on established biological principles
Technical and Research domain	Proteins secreted by cells are difficult to trace back to the donor	Unique to ViaD	The secreted proteins diffuse freely into the shared culture medium, and cannot be assigned to their donor using genetic demultiplexing approaches	Theoretical extrapolation based on established biological principles
Practical domain	Health concern for lab personnel due to exposure to pathogens	Amplified in ViaD	Even though conventional cell culture can also get infected by a pathogen, in ViaD, an infection in one line can contaminate all pooled lines, some of which may be highly permissive, increasing the risk of infection to lab personnel	(Weiskirchen et al., 2023) Theoretical extrapolation based on established biological principles
Practical domain	The heightened risk of cross-contamination of cell lines requires the disposal of the entire village	Amplified in ViaD	The risk of increased costs due to contamination is amplified as a single infected cell line can contaminate all lines	(Weiskirchen et al., 2023) Theoretical extrapolation based on established biological principles
Practical domain	Higher upfront costs may occur due to proliferation rate testing	Unique to ViaD	To prevent overrepresentation of fast-growing lines, initial testing of cell line growth rates is often recommended and can increase initial costs	Theoretical extrapolation based on established biological principles
Practical domain	Hurdles in adoption in some labs due to extensive expertise and infrastructure requirement	Amplified in ViaD	Data analysis in ViaD relies on single-cell approaches and complex bioinformatic pipelines and can render ViaD’s adoption more challenging in some labs compared to conventional cell culture	(Cuomo et al., 2020; Neavin et al., 2023; Wells et al., 2023)

*The platform-related issues arise from the multiplex nature of ViaD and cannot be completely addressed. As these issues stem from the design of ViaD itself, they are expected to exist across all applications.

**TABLE 3:** Overview of application-specific issues across domains.

Domain	Issue	Unique to ViaD/Amplified/Overlapped	Notes	References
Technical and Research domain	Not suitable for studying certain types of immune cells	Unique to ViaD	This issue is only relevant when ViaD is used for immunology-related applications, where immune cells from different donors can attack and kill each other, disrupting entire culture	(Mangelinck et al., 2023; Harper et al., 2015) Theoretical extrapolation based on established biological principles
Technical and Research domain	Limitations in co-culture studies	Unique to ViaD	Co-cultures can confound results in ViaD because cell type A from donor 1 can interact with cell type B from donor 2, and vice versa. This limitation is only relevant when ViaD is applied to biological questions that are critically dependent on the interactions between multiple cell types	(Chie et al., 2025; Vahsen et al., 2022) Theoretical extrapolation based on established biological principles
Practical domain	Increased cost risk in highly iterative studies	Amplified in ViaD	Although experimental failure can occur in any cell culture system, its impact is amplified in ViaD, where a failed condition can compromise the entire pooled culture thereby increasing costs	(Larsson et al., 2020; Sánchez-Díez et al., 2025) Theoretical extrapolation based on pooled culture design
Practical domain	Laboratory results partially capture physiology, risking poor clinical decisions	Overlapped with similar technologies	Concerns arise when ViaD is used to guide clinical decision-making. While this issue overlaps with conventional cell culture, it may become more prominent in ViaD due to its anticipated broader clinical implementation	(Centeno et al., 2018; McKenzie et al., 1995; Wang et al., 2024) Theoretical extrapolation based on established biological principles
Societal domain	Disclosure of genetic risk to the family despite patient’s refusal	Amplified in ViaD	Disclosure of incidental findings reflects a tension between patient consent and moral obligations to prevent harm to relatives. This issue may become amplified in ViaD due to accelerated functional interpretation of genetic variation	(Phillips et al., 2021) Theoretical extrapolation based on genetic privacy considerations
Societal domain	Liability for treatment decisions based on ViaD data	Overlapped with similar technologies	Liability for adverse treatment outcomes may become increasingly relevant as ViaD is expected to be implemented in for clinical applications	(Thorogood et al., 2017) Theoretical extrapolation based on clinical governance considerations

*The application-specific issues only arise when ViaD is used for particular applications and are insignificant for other uses.

### Platform-level issues (intrinsic to the ViaD design)

Within the technical and research domain, a central platform-level issue is the unequal representation of donor-derived cell lines during culture and differentiation (Table 2). Differences in proliferation, survival, stress tolerance, and donor-specific lineage commitment or maturation may cause certain donor lines to become overrepresented, potentially biasing population-level readouts (Cuomo et al., 2020; Neavin et al., 2023). Mitigation strategies such as pre-screening donor lines, constructing smaller “mini-villages” using cells from donors with similar proliferation rate, or limiting the proliferative phase (e.g., pooling lines only during the less proliferative stages such as during differentiation protocols) can reduce this risk of highly proliferative lines dominating the culture, but add experimental complexity.

Nevertheless, because ViaD enables simultaneous culture of many donor cell lines, a large number of lines can still be retrieved and analyzed even if a few less proliferative lines are lost. Hence, ViaD remains less problematic and provides a substantially higher throughput compared to conventional cell culture.

A closely related platform-level issue concerns inter-donor cell-cell signaling and interaction. In a shared culture environment, donor-specific secreted factors, metabolic competition, or stress responses can influence neighboring cells, complicating the attribution of observed phenotypes to genetic rather than environmental effects (Table 2). Although large-scale ViaD experiments may help distinguish systematic genetic effects from background noise, these interactions represent a persistent source of uncertainty that must be considered during experimental design and interpretation (Neavin et al., 2023).

The differential growth rates between cells and inter-donor cell-cell signaling and interaction also occur in conventional single-donor cultures. However, in conventional cell culture, paracrine signaling occurs between cells from the same donor and is therefore interpreted as an intrinsic feature of that individual. Likewise, differences in cell growth rates are confined to independently cultured donor lines, so they do not alter the representation of other donors or bias comparative analyses. In ViaD, inter-donor paracrine signaling may introduce interactions that do not reflect the physiological context being modelled and can complicate interpretation of gene expression readouts. Similarly, differential proliferation can alter donor representation within the pooled culture, causing slow proliferating donor lines to be lost or masked and potentially biasing population-level conclusions.

A distinct issue relates to donor identification through genetic demultiplexing. ViaD relies on naturally occurring genetic variation to assign cells to their donor of origin. As a result, cell lines that share highly similar or identical genetic backgrounds, such as multiple iPSC clones from the same donor, isogenic pairs (including gene-corrected or CRISPR-edited derivatives), or monozygotic twin-derived cell lines, may not be distinguishable using standard genotype-based demultiplexing approaches (Cuomo et al., 2020). In such cases, additional labeling strategies, including genetic barcoding, cell hashing, or fluorescent reporters, may be required to enable accurate cell identification (Table 2).

Moreover, a platform-level limitation relates to molecular readouts. While single-cell transcriptomic data can be reliably demultiplexed to individual donors (Cuomo et al., 2020; Neavin et al., 2023; Wells et al., 2023), proteins secreted into the culture medium and other extracellular signals cannot be easily traced back to their secreting cell because they lack the nucleotide-level variant information required for routine genetic demultiplexing. Unlike conventional cell culture, where secreted proteins can be attributed to the sole donor by design, ViaD pools cells from many donors in a shared extracellular environment, making donor-specific attribution of secreted proteins inherently challenging. This limitation constrains the interpretability of certain functional assays and applies across all ViaD applications, making it an intrinsic platform-level issue (Table 2).

For studies where secretome analysis is central, using conventional, single-donor cell cultures is recommended as cells are cultured and analyzed separately for each donor, so assigning cells or their secreted proteins back to a donor of origin is straightforward without any ambiguity.

Beyond biological considerations, ViaD also presents organizational, costs and safety-related issues at the platform level. ViaD implementation requires coordinated expertise in wet-lab biology, single-cell sequencing, and bioinformatics, as well as access to specialized infrastructure. Data analysis in ViaD relies on single-cell sequencing approaches and complex bioinformatic pipelines for donor demultiplexing and interpretation of pooled datasets. While similar technologies can also be applied to conventional cell culture systems, they are not always required, as analyses can often be performed using less sophisticated approaches such as bulk sequencing or RT-qPCR.

In addition, pooled cultures may be more vulnerable to contamination events, as contamination originating from a single donor line can spread throughout the entire culture, and certain lines may be highly permissive to pathogens, potentially increasing the exposure risk to lab personnel (Weiskirchen et al., 2023). This amplifies both biosafety risks and the potential for financial losses due to the loss of the entire culture, relative to conventional cell culture (Table 2).

Rigorous quality control measures, including regular sterility testing, careful donor line screening prior to pooling, and strict adherence to aseptic techniques can minimize the risk of contamination and exposure to pathogens.

### Application-specific issues (dependent on experimental context)

In contrast to platform-level constraints, application-specific issues arise only when ViaD is used for particular biological or translational purposes and are therefore not universally applicable (Table 3).

One prominent example concerns applications involving certain immune cell types. In pooled cultures, donor-specific immune recognition mechanisms may trigger immune activation or cell death analogous to allograft rejection (Mangelinck et al., 2023; Harper et al., 2015). Because such immune interactions do not occur under normal physiological conditions, they may limit the suitability of ViaD for certain immunological studies. This issue is specific to immune-related applications and is therefore classified as application-specific (Table 3).

Similarly, co-culture studies that rely on tightly controlled interactions between defined cell types may be difficult to interpret when cells originate from multiple donors. For instance, in neurodegenerative disease research, interactions between neurons, oligodendrocytes, and microglia are often central to the biological question (Chie et al., 2025; Vahsen et al., 2022). In a ViaD setting, cross-donor interactions between these cell types can obscure causal genotype-phenotype relationships, making this limitation relevant only for specific co-culture applications rather than for the platform as a whole (Table 3).

Unlike most other issues, immune-related and co-culture limitations are unlikely to be mitigated through technical optimization. Therefore, conventional cell culture could be better suited for such applications.

Cost and scalability considerations vary by application. While ViaD can reduce per-sample costs in large-scale experiments, upfront setup costs, single cell analysis requirements, and the risk of whole-culture loss due to experimental error may require extra caution in applications requiring repeated iteration. Drug sensitivity screening (e.g., IC50 determination) is one such example that requires testing multiple drug concentrations across repeated iterations each carrying risk of whole-culture loss due to cytotoxicity (Larsson et al., 2020; Sánchez-Díez et al., 2025).

Although repeated testing is an inherent requirement of drug sensitivity screening, it can substantially increase the costs in ViaD compared with conventional cell culture, where a failed condition results in the loss of a single cell line rather than disrupting the entire village culture (Table 3). Because this risk cannot be fully eliminated through experimental optimization, researchers considering ViaD for this type of screening should consider this added cost risk into their study design.

Clinical application-specific issues arise when ViaD data are used to inform downstream decision-making, such as drug response prediction and functional precision medicine. In such contexts, the incomplete representation of physiological complexity, such as the absence of systemic immune, endocrine, or metabolic interactions, may limit predictive validity of ViaD (Alexander et al., 2020; Wang et al., 2024). This limitation overlaps with other *in vitro* models and may influence a range of ViaD applications. However, this may become particularly relevant if ViaD results are used to inform clinical decisions, as its scalability and potential cost advantages could support its future application as a functional precision medicine test. This justifies its classification as an application-specific issue that overlaps with conventional cell culture (Table 3).

Societal considerations further illustrate the application-dependent nature of some issues. Although data governance is relevant to all ViaD uses, issues such as handling of incidental findings may become more pronounced in applications involving clinically relevant data. For example, incidental findings in clinical contexts can reveal the potentially harmful clinical significance of a genetic mutation; such findings cannot be disclosed without the patient’s consent. However, there could be exceptions to the privacy rule when there is a serious and imminent risk to the health of biological relatives, as they may carry the same mutation (Phillips et al., 2021). While such incidental findings could also occur in conventional cell culture, given ViaD ability to accelerate the understanding of the functional significance of genetic mutations, this issue may become amplified in ViaD (Table 3).

Moreover, questions of liability for treatment outcomes may arise primarily in clinical decision-making contexts where ViaD generated data are used to inform patient care (Thorogood et al., 2017). Therefore, these issues depend on the intended use of ViaD rather than on its core design. While this issue would also occur in conventional cell culture, it is more likely to be encountered with ViaD due to the anticipated broader use of ViaD as a functional precision medicine test (Table 3).

## Conclusion

Emerging multiplex cell culture platforms, such as ViaD, offer promising and unique opportunities to perform human genetic studies with functional readouts, and disease-relevant biological processes at the population scale. However, the multiplex design of ViaD also introduces specific issues and limitations that are either absent or much less pronounced in conventional single-donor cultures.

In this review, we used a structured, domain-based framework as a tool to identify those issues. By systematically mapping the issues and limitations across various domains, and by explicitly distinguishing between platform-level and application-specific constraints, we provide a nuanced account of how the strengths and limitations of ViaD depend on context.

In summary, ViaD enables population-scale functional studies of human genetic variation at a scale that was unfeasible with conventional cell culture. However, its multiplex design introduces a range of platform-related and application-specific issues that can influence experimental feasibility, data interpretation, costs, and the suitability of the platform for different biological questions (Figure 2). By systematically identifying and categorizing these challenges, this review provides researchers with a practical framework to anticipate potential sources of bias, unexpected costs and technical complexity, as well as to recognize applications where alternative methods may be better suited. These insights can facilitate more informed decisions during study design and determine when ViaD is likely to offer meaningful advantages over conventional cell culture approaches.

While this review focuses on ViaD, the insights extend more broadly to other emerging *in vitro* and platform technologies that combine scale, technical complexity, and multiple potential applications. As biological research increasingly relies on such platforms, structured approaches that integrate technical and research, practical and societal considerations will become increasingly important for responsible innovation and robust interpretation of experimental findings.

Future work should build on this conceptual foundation by empirically studying how different platform-level issues manifest across applications and by developing practical guidance for integrating multiplex cell culture platforms into experimental pipelines. Ultimately, combining technological innovation with structured reflection can help ensure that new *in vitro* platforms realize their scientific potential while avoiding misapplication and overinterpretation.

## References

[B1] Alexander L. Baazim H. Bergthaler A. (2020). Systemic immunometabolism: challenges and opportunities. Immunity 53 (3), 496–509. 10.1016/j.immuni.2020.08.012 32937151 PMC7491485

[B2] Boyle E. A. Li Y. I. Pritchard J. K. (2017). An expanded view of complex traits: from polygenic to omnigenic. Cell 169 (7), 1177–1186. 10.1016/j.cell.2017.05.038 28622505 PMC5536862

[B3] Cederquist G. Y. Tchieu J. Callahan S. J. Ramnarine K. Ryan S. Zhang C. (2020). A Multiplex Human Pluripotent Stem Cell Platform Defines Molecular and Functional Subclasses of autism-related Genes. Elsevier BV. 10.1016/j.stem.2020.06.004 PMC7376579 32619517

[B4] Centeno E. G. Z. Cimarosti H. Bithell A. (2018). 2D versus 3D human induced pluripotent stem cell-derived cultures for neurodegenerative disease modelling. Mol. Neurodegener. 13 (1), 27. 10.1186/s13024-018-0258-4 29788997 PMC5964712

[B5] Cerneckis J. Cai H. Shi Y. (2024). Induced pluripotent stem cells (iPSCs): molecular mechanisms of induction and applications. Signal Transduct. Target. Ther. 9 (1), 112–126. 10.1038/s41392-024-01809-0 38670977 PMC11053163

[B6] Chie S. Szentpetery Z. Generali M. Kuhlmann T. Natalucci G. Miletta M. (2025). Human iPSC-derived neuron and oligodendrocyte co-culture as a small-molecule screening assay for myelination. Bio-Protocol 15 (1366), e5227. 10.21769/BioProtoc.5227 40364981 PMC12067306

[B7] Cuomo A. S. E. Seaton D. D. Mccarthy D. J. Martinez I. Bonder M. J. Garcia-Bernardo J. (2020). Single-cell RNA-sequencing of differentiating iPS cells reveals dynamic genetic effects on gene expression. Nat. Commun. 11 (1), 810. 10.1038/s41467-020-14457-z 32041960 PMC7010688

[B8] Farbehi N. Neavin D. R. Cuomo A. S. E. Studer L. MacArthur D. G. Powell J. E. (2024). Integrating population genetics, stem cell biology and cellular genomics to study complex human diseases. Nat. Genet. 56 (5), 758–766. 10.1038/s41588-024-01731-9 38741017

[B9] Ghosh S. Nehme R. Barrett L. E. (2022). Greater genetic diversity is needed in human pluripotent stem cell models. Nat. Commun. 13 (1), 7301–7307. 10.1038/s41467-022-34940-z 36435871 PMC9701202

[B10] Grant Rowe R. Daley G. Q. (2019). Induced pluripotent stem cells in disease modelling and drug discovery. Nat. Rev. Genet. 20 (7), 377–388. 10.1038/s41576-019-0100-z 30737492 PMC6584039

[B13] Harper S. J. F. Ali J. M. Wlodek E. Negus M. C. Harper I. G. Chhabra M. (2015). CD8 T-cell recognition of acquired alloantigen promotes acute allograft rejection. Proc. Natl. Acad. Sci. 112 (41), 12788–12793. 10.1073/pnas.1513533112 26420874 PMC4611606

[B15] Jerber J. Seaton D. D. Cuomo A. S. E. Kumasaka N. Haldane J. Steer J. (2021). Population-scale single-cell RNA-seq profiling across dopaminergic neuron differentiation. Nat. Genet. 53 (3), 304–312. 10.1038/s41588-021-00801-6 33664506 PMC7610897

[B16] Kilpinen H. Goncalves A. Leha A. Afzal V. Alasoo K. Ashford S. (2017). Common genetic variation drives molecular heterogeneity in human iPSCs. Nature 546 (7658), 370–375. 10.1038/nature22403 28489815 PMC5524171

[B17] Kristensen F. B. Lampe K. Wild C. Cerbo M. Goettsch W. Becla L. (2017). The HTA core Model®—10 years of developing an international framework to share multidimensional value assessment. Value Health 20 (2), 244–250. 10.1016/j.jval.2016.12.010 28237203

[B19] Larsson P. Engqvist H. Biermann J. Werner Rönnerman E. Forssell-Aronsson E. Kovács A. (2020). Optimization of cell viability assays to improve replicability and reproducibility of cancer drug sensitivity screens. Sci. Rep. 10 (1), 5798. 10.1038/s41598-020-62848-5 32242081 PMC7118156

[B21] Maarten J. I. J. Steuten L. M. G. (2011). Early assessment of medical technologies to inform product development and market access. Appl. Health Econ. Health Policy 9 (5), 331–347. 10.2165/11593380-000000000-00000 21875163

[B22] Mangelinck A. Dubuisson A. Becht E. Dromaint-Catesson S. Fasquel M. Provost N. (2023). Characterization of CD4+ and CD8+ T cells responses in the mixed lymphocyte reaction by flow cytometry and single cell RNA sequencing. Front. Immunol. 14, 1320481. 10.3389/fimmu.2023.1320481 38283342 PMC10820991

[B23] McKenzie R. Fried M. W. Sallie R. Conjeevaram H. Di Bisceglie A. M. Park Y. (1995). Hepatic failure and lactic acidosis due to fialuridine (FIAU), an investigational nucleoside analogue for chronic hepatitis B. N. Engl. J. Med. 333 (17), 1099–1105. 10.1056/NEJM199510263331702 7565947

[B24] Mitchell J. M. Nemesh J. Ghosh S. Handsaker R. E. Mello C. J. Meyer D. (2020). Mapping Genetic Effects on Cellular Phenotypes with “Cell Villages”. 10.1101/2020.06.29.174383

[B25] Neavin D. R. Steinmann A. M. Farbehi N. Chiu H. S. Daniszewski M. S. Arora H. (2023). A village in a dish model system for population-scale hiPSC studies. Nat. Commun. 14 (1), 3240. 10.1038/s41467-023-38704-1 37296104 PMC10256711

[B26] O'Rourke B. Oortwijn W. Schuller T. International Joint Task Group (2020). The new definition of health technology assessment: a milestone in international collaboration. Int. J. Technol. Assess. Health Care 36 (3), 187–190. 10.1017/S0266462320000215 32398176

[B27] Panopoulos A. D. D'Antonio M. Benaglio P. Williams R. Hashem S. I. Schuldt B. M. (2017). iPSCORE: a resource of 222 iPSC lines enabling functional characterization of genetic variation across a variety of cell types. Stem Cell Rep. 8 (4), 1086–1100. 10.1016/j.stemcr.2017.03.012 PMC5390244 28410642

[B28] Phillips A. Borry P. Van Hoyweghen I. Vears D. F. (2021). Disclosure of genetic information to family members: a systematic review of normative documents. Genet. Med. 23 (11), 2038–2046. 10.1038/s41436-021-01248-0 34234303

[B29] Sánchez-Díez M. Romero-Jiménez P. Alegría-Aravena N. Gavira-O’Neill C. E. Vicente-García E. Quiroz-Troncoso J. (2025). Assessment of cell viability in drug therapy: IC50 and other new time-independent indices for evaluating chemotherapy efficacy. Pharmaceutics 17 (2), 247. 10.3390/pharmaceutics17020247 40006615 PMC11859577

[B30] Spitalieri P. Talarico V. R. Murdocca M. Novelli G. Sangiuolo F. (2016). Human induced pluripotent stem cells for monogenic disease modelling and therapy. World J. Stem Cells 8 (4), 118–135. 10.4252/wjsc.v8.i4.118 27114745 PMC4835672

[B31] Takahashi K. Tanabe K. Ohnuki M. Narita M. Ichisaka T. Tomoda K. (2007). Induction of pluripotent stem cells from adult human fibroblasts by defined factors. Cell 131 (5), 1–12. 10.1016/j.cell.2007.11.019 18035408

[B32] Thorogood A. Cook-Deegan R. Knoppers B. M. (2017). Public variant databases: liability? Genet. Med. 19 (7), 838–841. 10.1038/gim.2016.189 27977006 PMC5527130

[B33] Vahsen B. F. Gray E. Candalija A. Cramb K. M. L. Scaber J. Dafinca R. (2022). Human iPSC co-culture model to investigate the interaction between microglia and motor neurons. Sci. Rep. 12 (1), 12606–12617. 10.1038/s41598-022-16896-8 35871163 PMC9308778

[B34] van Zanten E. S. Loehrer E. A. van Meurs J. B. J. Narcisi R. Gribnau J. H. Poot R. A. (2026). Bridging population and cell: modelling complex diseases with human induced pluripotent stem cells. Eur. J. Hum. Genet. EJHG 34, 741–748. 10.1038/s41431-026-02071-4 41917264 PMC13247087

[B35] Wang L. Hu D. Xu J. Hu J. Wang Y. (2024). Complex *in vitro* model: a transformative model in drug development and precision medicine. Clin. Transl. Sci. 17 (2), e13695. n/a. 10.1111/cts.13695 38062923 PMC10828975

[B36] Weiskirchen S. Schröder S. K. Buhl E. M. Weiskirchen R. (2023). A beginner’s guide to cell culture: practical advice for preventing needless problems. Cells 12 (5), 682. 10.3390/cells12050682 36899818 PMC10000895

[B37] Wells M. F. Nemesh J. Ghosh S. Mitchell J. M. Salick M. R. Mello C. J. (2023). Natural variation in gene expression and viral susceptibility revealed by neural progenitor cell villages. Cell Stem Cell 30 (3), 312–332.e13. 10.1016/j.stem.2023.01.010 36796362 PMC10581885

[B38] Yamanaka S. (2026). Two decades of induced pluripotent stem cell research: from discovery to diverse applications. Cell Stem Cell 33 (3), 372–381. 10.1016/j.stem.2026.02.003 41795426

